# Immortal time bias in retrospective analysis: comment on ‘Efficacy and safety of long-term treatment with lenalidomide and dexamethasone in patients with relapsed/refractory multiple myeloma'

**DOI:** 10.1038/bcj.2015.4

**Published:** 2015-02-27

**Authors:** P-Y Hsieh, C-J Liu, C-J Teng

**Affiliations:** 1 Division of Oncology and Hematology, Department of Medicine, Far Eastern Memorial Hospital, New Taipei City, Taiwan; 2 School of Medicine, National Yang-Ming University, Taipei, Taiwan; 3 Institute of Public Health, National Yang-Ming University, Taipei, Taiwan; 4 Division of Hematology and Oncology, Department of Medicine, Taipei Veterans General Hospital, Taipei, Taiwan

We read with interest ‘Efficacy and safety of long-term treatment with lenalidomide and dexamethasone in patients with relapsed/refractory multiple myeloma'.^1^ Dimopoulos *et al.* reported a retrospective post-hoc pooled analysis by using the data from two randomized trials, MM-009 and MM-010, which showed improved outcomes in patients with relapsed/refractory multiple myeloma treated with lenalidomide and dexamethasone. An important finding in Dimopoulos's report is the association between humoral improvement (uninvolved immunoglobulin A) and the long-term benefit of therapy. It shows humoral responders had better progression-free survival and overall survival compared with humoral nonresponders, in Figure 2. However, humoral response could occur at anytime from cycle one to ten. If the data was analyzed by using a log-rank test or time-independent Cox regression model, it is likely that an ‘immortal time bias' could interfere with this result.^2, 3, 4, 5^

‘Immortal time' refers to a time interval during the follow-up period in which the outcome could never occur because of exposure definition. For example, death cannot occur in the first 5 months of follow-up, in patients who had humoral response after at least six cycles (of each 28-day cycle). In conventional survival analysis, the immortal time bias can confer a spurious survival advantage to the responder group if the response is measured in the middle of the follow-up period but not at the beginning of the trial. Humoral improvement might predict the outcomes of the patients with multiple myeloma. Nevertheless, the result should be analyzed using time-dependent analysis,^2, 5^ such as Cox regression with response status as a time-dependent covariate. We look forward to see such reanalysis of the original data.
